# Impact of the Family Environment on Juvenile Mental Health: eSports Online Game Addiction and Delinquency

**DOI:** 10.3390/ijerph15122850

**Published:** 2018-12-13

**Authors:** Chulhwan Choi, Mary A. Hums, Chul-Ho Bum

**Affiliations:** 1 Department of Golf Industry, College of Physical Education, Kyung Hee University, Seocheon-dong 1, Giheung-gu, Yongin-si, Gyeonggi-do 17104, Korea; chulhwanchoi@yahoo.com; 2 Department of Health and Sport Science, University of Louisville, SAC East 104Q, University of Louisville, Louisville, KY 40292, USA; mhums@louisville.edu; 3 Department of Sports Taekwondo, Graduate School of Physical Education, Kyung Hee University, Seocheon-dong 1, Giheung-gu, Yongin-si, Gyeonggi-do 17104, Korea

**Keywords:** eSports, family form, game addiction, juvenile delinquency, participation

## Abstract

Family types in Asian countries are rapidly changing as the society is changing. Thus, in this study, we analyzed and compared how the newly evolving family types (multicultural/dual-income) affect adolescents’ online game addiction, delinquency, and online gaming (eSports) participation motivation. Multiple regression analysis was performed to examine the causal relationships between the variables, and multivariate analysis of variance and analysis of variance were performed for comparative analyses. The results indicate that adolescents from dual-income families scored significantly higher on all factors related to juvenile delinquency and addiction factors (“salience”, “tolerance” and “withdrawal”). Additionally, adolescents from multicultural families revealed significantly higher scores on an addiction factor, “mood modification”. Lastly, adolescents in dual-income families were motivated to play online games to pass the time, and adolescents in multicultural families play online games to engage in social interaction. Results of this study may provide the answers required to help address societal issues related to adolescents in a changing society.

## 1. Introduction

In Asian countries in this age, significant changes are occurring in the existing family system. For example, the entry of foreign migrant workers and an increase of international marriages have led to a shift from a traditional “single ethnic society” to a “multicultural society” [1]. According to the South Korean Ministry of the Interior and Safety [2], one out of 10 new marriages is international. This type of multicultural family is defined as a family composed of members belonging to different ethnicities or cultural backgrounds [3].

Moon et al. [4] have suggested that multicultural families are, unfortunately, experiencing financial difficulties, reaching only 59% of the income earned in other households. In particular, these authors found that the lower the economic level, the higher the probability that both husband and wife worked, with most couples working long hours for low wages.

Although such dual-income couples can be a positive factor in financial terms, considerable problems have been found to occur in the home [5]. In dual-income households, both husband and wife have jobs and must simultaneously perform the role of worker, spouse, and parent [6]. In this respect, Peterson and Hennon [7] pointed out the major challenges that dual-income couples experience include role overload from dealing with work and family at the same time and, at a deeper level, difficulties with parenting.

Previous research has found that children of dual-career parents who work long hours generally spend a great amount of time watching TV after school, using the internet, and playing online games [8]. Compared to adults, adolescents in particular are more likely to be addicted to online games as they consist of content that can stimulate curiosity and competition [9]. Ultimately, adolescents’ addiction to online games has been shown to have a major impact on their lives [10,11,12,13]. In particular, adolescents addicted to online games tend to exhibit behaviors related to juvenile delinquency, such as drinking and smoking, and tend to hang out with friends who are also addicted [14].

With the high penetration rate of PCs as well as the expansion of high-speed information/communication networks in recent years, modern humans in developed countries live in an environment where they can easily access the internet [15]. In particular, as cutting-edge technology began to influence the electronics industry, electronic sports, or eSports for short, which are video games played in a virtual space, have dramatically increased in popularity [16,17].

Therefore, the study examined which online game addiction factors drive juvenile delinquency. Additionally, the present study investigated the effects of the changed family form (e.g., single-income/dual-income households and multicultural/monocultural families) on eSports game addiction, delinquency, and eSports participation motivation.

## 2. Literature Review 

### 2.1. Online Game Addiction

As the social issue of online game addiction emerged with the growing popularity of eSports [18], playing online games became negatively perceived, similar to gambling addiction or alcoholism [19]. The definition of game addiction refers to an “excessive and compulsive use of video games that results in social and/or emotional problems; despite these problems, the gamer is unable to control this excessive use” [20] (p. 78). In other words, online game addiction can be determined based on how much one depends psychologically or emotionally on online games. Most previous studies have claimed that mental illness [21,22,23,24,25,26,27,28] and inactive lifestyle [29] are the consequences of game addiction. The problems mentioned above are also found in adolescents, and their excessive online gaming has been discussed from a negative perspective [30].

### 2.2. Juvenile Delinquency

In general, the consistent opinions, attitudes, and behaviors that are expected by society are called social norms, and any behavior that deviates from these norms are considered deviant behaviors. In other words, when adolescents take part in delinquent behaviors that deviate from social norms, this is explained as juvenile delinquency [31]. Since there is no consistent definition of juvenile delinquency in previous studies, the majority of studies, including the present study, focus on the deviant behaviors themselves rather than the actual person. Drinking, smoking, and unauthorized absenteeism are the main types of delinquent behaviors among adolescents. While these behaviors are not considered problematic for adults, they are considered delinquent based on their status as adolescents [32]. Because drinking and smoking behaviors tend to decrease naturally over the course of adulthood, some studies see these behaviors as normal behaviors with transitional traits [33]. On the other hand, according to some studies, it is difficult to rule out the potential for these adolescents to become criminals as adults [34]. Lastly, unauthorized absenteeism often occurs due to stress from academics or family matters [35], and repeated unauthorized absenteeism may develop into more serious delinquency or crimes in the future [31].

“Adolescents are confronted with a variety of cumulated stressors such as physical and hormonal changes, as well as shifts in personal value and belief systems” ([36], p. 3–4). Research has shown that online games are used as a tool for coping with such stress, and that addiction to online games leads to behavioral issues in adolescents, including displays of aggressive tendencies and a decrease in self-control [37]. While some researchers argue that excessive online gaming is strongly associated with adolescent misconduct [38], others maintain that the evidence of such relationship is not only weak, but almost nonexistent [39,40].

### 2.3. Participation Motivation in eSports

Motivation plays a critical role in understanding consumer behavior. It is defined as the factor that influences people’s unconscious determination of behavior in a given situation [41]. Research on sport participation motivation explores why consumers participate in sports and what benefits they gain from participation [42]. The reasons why people participate in sports are determined by the active choice to satisfy their needs, and hence, uses and gratification theory has been utilized as a basic theoretical framework for explaining people’s propensity to participate [43].

As the number of eSports fans has now exceeded 205 million worldwide [44], it is interesting to track participation in eSports as well. However, as motivation to participate in eSports tends to be different from participation in general sports activities, it is not appropriate to apply participation motivation in traditional sports to the understanding of eSports [45]. In this context, Lee and Schoenstedt [45] developed 14 eSports participation motives from modifying and supplementing participation motivation factors of existing general sports, such as baseball, that are relevant to eSports (Social Interaction, Fantasy, Identification with Sport, Diversion, Competition, Entertainment, Sport Knowledge Application, Arousal, Design/Graphics, Pass Time, Control, Skill Building for Playing Actual Sport, Permanence, and Peer Pressure). Comparative analysis with traditional sport consumption motives revealed the significant influence of three factors (i.e., competition, peer pressure, and skill building for playing actual sport).

### 2.4. Significance of the Study and Research Questions

Family types are changing in Asian countries to accommodate diverse cultures and evolving environments. The purpose of this study was to examine how new family types in our Korean society (multicultural families and dual-income families) affect adolescent behavior. Specifically, this research focused on: (a) online game addiction (b) juvenile delinquency, (c) influence of online game addiction on juvenile delinquency, and (d) eSports participation motivation. Participants consisted of four groups: (a) monocultural, single-income families, (b) monocultural, dual-income families, (c) multicultural, single-income families, and (d) multicultural, dual-income families. Through this study, we will be able to interpret adolescents’ online game addiction and delinquency from the perspective of changing family types, while presenting solutions to the problem. This study will be guided by the following research questions:

RQ1. What online game addiction factors drive juvenile delinquency?
RQ2. What are the differences in online game addiction, juvenile delinquency, and eSports participation motivation among the forms of families?

## 3. Materials and Methods

### 3.1. Participants

Much like in Western countries, multicultural families have become more common in Asian countries due to the increased inflow of foreigners following changes in the social flow [1]. Unfortunately, most of these families experience financial difficulties, and both men and women work full time [3]. This has been reported to potentially have a considerable impact on how children are raised [5]. Hence, in order to analyze these social phenomena, adolescents were selected as the subject of this study. Adolescence in this study refers, as is typical, to the ages of 13 to 18 [46,47]. Although there are cultural differences [47], people in this age group mostly include students in middle and high school. Thus, in light of Korean culture, this study limited the age of participants to 12–18, to coincide with middle and high school in Korea.

The participants selected for this study were 12 to 18-year-old adolescents from either monocultural or multicultural families. A pilot test was conducted to modify sentences and word choice that might have been somewhat difficult for adolescents to understand. Following the research aim, a purposive sampling method was employed at two middle/high schools in southern Gyeonggi Province during five multicultural family day events, from the second half of 2017 to the first half of 2018, with the help of multicultural support centers operated by the city or county. Participation in the survey was voluntary, and each questionnaire was completed using the self-administered method. Through these procedures, a total of 300 questionnaires were distributed, of which 268 were returned (response rate approximately 89%). After excluding 22 unfinished questionnaires, the final 246 surveys were used for analysis.

### 3.2. Measures

#### 3.2.1. Online Game Addiction

This measure consisted of factors identified by Lemmens et al. [20] to assess game addiction in adolescents, including salience (e.g., “Have you felt addicted to eSports?”), tolerance (e.g., “Did you play longer than intended?”), mood modification (e.g., “Did you play eSports to forget about real life?”), relapse (e.g., “Were you unable to reduce time to play eSports?”), withdrawal (e.g., “Have you felt bad when you were unable to play?”), conflict (e.g., “Did you have fights with others over your time spent on eSports?”), and problem (e.g., “Did you feel bad after playing for a long time?”). There are 21 items, showing acceptable Cronbach’s alphas ranging from 0.84 to 0.90 in the previous study, in total with three items per factor utilizing 5-point Likert scale with 1 point for “Strongly disagree”, 2 for “Disagree”, 3 for “Neither agree nor disagree”, 4 for “Agree”, and 5 for “Strongly agree”.

#### 3.2.2. Juvenile Delinquents

Three items used by Lee [31] were modified and utilized in this measure asking if one has engaged in delinquent behaviors (smoking, *α* = 0.82; drinking, *α* = 0.89; school absence, *α* = 0.87 in the previous study) during the past year. This study employed a 5-point Likert scale with 1 point for “Never”, 2 for “Rarely”, 3 for “Sometimes”, 4 for “Often”, and 5 for “Very often”.

#### 3.2.3. eSports Participation Motivation

Based on various previous studies [48,49], the present study used eight factors regarding motivation to participate in eSports, which were used by Lee and Schoenstedt showing acceptable Cronbach alphas ranging from 0.71 to 0.86 [45]. These factors include entertainment (e.g., “I play eSport because it is enjoyable”), competition (e.g., “I like to play to prove to others that I am the best”), permanence (e.g., “I tend to play eSport because they are readily available”), pass time (e.g., “I often play eSport because there is nothing else to do”), social interaction (e.g., “I use eSport as a reason to get together with others”), diversion (e.g., “Playing eSport gives me a break from my regular routine”), arousal (e.g., “I play eSport because they excite me”), and peer pressure (e.g., “My friends force me to play eSport”). Social interaction and arousal each consisted of four items, while the other factors consisted of three items each, for a total of 26 items applying 7-point Likert scale with 1 point for “Strongly disagree”, 2 for “Disagree”, 3 for “Somewhat disagree”, 4 for “Neutral”, 5 for “Somewhat agree”, 6 for “Agree”, and 7 for “Strongly agree”.

### 3.3. Data Analysis

In a confirmatory factor analysis (CFA), Comparative fit index (CFI), Tucker-Lewis index (TLI), root mean square residual (RMR), and root mean square error of approximation (RMSEA) were used to test model fitness. In addition, reliability analysis was conducted using Cronbach’s alpha to test the internal consistency of the items. Multivariate analysis of variance (MANOVA) including post-hoc analyses were performed to compare and analyze the differences in online game addiction, juvenile delinquency, and participation motivation among adolescents across the four groups. Additionally, multiple regression analysis was conducted to examine the causal relationships between variables.

## 4. Results

### 4.1. Socio-Demographic Statistics

A total of 246 questionnaires were utilized, consisting of responses from 202 boys (82%) and 44 girls (18%). Participation was limited to adolescents of ages 12 to 18 (*M* = 14.40, *SD* = 1.924), fitting the goal of the research study. Family types could be divided into monocultural (*n* = 131, 53%) and multicultural (*n* = 115, 47%) families, as well as single-income (*n* = 133, 54%) and dual-income (*n* = 113, 46%) families. Finally, the four family-type groups that participated in the study were (a) monocultural/single-income (*n* = 70, 28%), (b) monocultural/dual-income (*n* = 61, 25%), (c) multicultural/single-income (*n* = 63, 26%), and multicultural/dual-income (*n* = 52, 21%) (Table 1).

### 4.2. Scale Validity and Reliability

From the results of the confirmatory factor analysis, all critical ratios in the regression weights were found to be greater than ±1.96. In addition, the values of squared multiple correlations (SMC) for all items were found to be greater than 0.4. In general, a SMC value closer to 1 explains the variance of the measured variables Tests of model fit were: Chi-square = 1145.097, DF = 1055, *p* = 0.027, Chi-square/DF = 1.095, CFI = 0.988, TLI = 0.986, RMR = 0.064, and RMSEA = 0.019. The model is considered to be a good fit if CFI and TLI values exceeded the criterion of 0.900, and RMR and RMSEA values are conservatively smaller than 0.05. As shown above, the results of this study mostly satisfy these figures, and thus this model is a fit.

Lastly, all Cronbach’s alpha showed good internal consistency for reliability based on the 0.70 cutoff [50]: (a) online game addiction (Salience, *α* = 0.923; Tolerance, *α* = 0.891; Mood Modification, *α* = 0.873; Relapse, *α* = 0.888; Withdrawal, *α* = 0.848; Conflict, *α* = 0.858; Problem, *α* = 0.823), (b) juvenile delinquency (*α* = 0.901), and **(c)** participation motivation (Entertainment, *α* = 0.854; Competition, *α* = 0.840; Permanence, *α* = 0.894; To pass time, *α* = 0.919; Social interaction, *α* = 0.848; Diversion, *α* = 0.887; Arousal, *α* = 0.902; Peer pressure, *α* = 0.900).

### 4.3. Multiple Regression Analysis

Multiple regression analysis resulted in a regression model where *F* = 8.559, *p* = 0.000, and *R*^2^ = 0.201, explaining 20.1% of the variance. More specifically, in adolescent online game addiction, salience (*t* = 5.24, *p* = 0.000), withdrawal (*t* = 3.02, *p* = 0.003), and tolerance (*t* = 2.01, *p* = 0.045) were found to increase delinquent behavior, while the other subfactors were not statistically significant (Table 2).

### 4.4. Multivariate Analysis of Variance (MANOVA) for Online Game Addiction

The multivariate test indicated statistically significant differences based on the forms of family on the eSports addiction [Wilks’ lambda = 0.460, *F*(21, 678.215) = 10.044, *p* = 0.00, partial *η*^2^ = 0.228]. Based on adjusted alpha level using Bonferroni correction (*p* = 0.05/7 = 0.007), univariate tests for (a) Salience, (b) Tolerance, (c) Mood Modification, (d) Withdrawal, and (e) Problem were statistically significant. However, the rest of the tests were not statistically significant: (a) Relapse and (b) Conflict. Results are shown in Table 3.

Follow-up Tukey post hoc analyses revealed that salience, tolerance, and withdrawal scores were higher in the two dual-income family groups than in the two single-income family groups. The mood modification score was higher in the two multi-cultural family groups than in the two monocultural family groups. Lastly, the problem score was significantly lower in the monocultural/single-income family group compared to the rest of the groups (Table 4).

### 4.5. Analysis of Variance (ANOVA) for Juvenile Delinquency

There was a statistically significant difference among the four groups on juvenile delinquency [*F*(3,242) = 48.100, *p* = 0.000]. A Tukey post hoc test found that the two dual-income family groups scored higher on measures of juvenile delinquency than the two single-income family groups. There was no statistically significant difference between monocultural and multicultural families (*p* > 0.05). More detailed information is shown in Table 5.

### 4.6. Multivariate Analysis of Variance (MANOVA) for Participation Motivation

The multivariate test revealed statistically significant differences based on the forms of family on eSports participation motivation [Wilks’ lambda = 0.445, *F*(24, 682.173) = 9.154, *p* = 0.00, partial *η*^2^ = 0.237]. Based on adjusted alpha level using Bonferroni correction (*P* = 0.05/8 = 0.006), univariate tests for (a) To pass time and (b) Social interaction were statistically significant. However, the rest of the tests were not statistically significant: (a) Entertainment, (b) Competition, (c) Permanence, (d) Diversion, (e) Arousal, and (f) Peer pressure as shown in Table 6.

Follow-up Tukey post hoc analyses revealed that the two dual-income family groups scored higher on “pass time” than the two single-income family groups, and the two multicultural family groups scored higher on “social interaction” than the two monocultural family groups (Table 7).

## 5. Discussion

### 5.1. Online Game Addiction due to Differences in Family Environment

In this study, the family type was found to have a significant effect on online game addiction. More specifically, “salience”, “tolerance”, and “withdrawal” scores were higher among adolescents from dual-income families than those from single-income families. This shows that, for adolescents from dual-income families whose parents have relatively little control [14], engagement in online games lasts longer, due to being difficult to self-control, which can lead to an unstable psychological state when gaming ceased. In addition, aside from single-income and dual-income families, the “mood modification” score was higher for adolescents from multicultural families than those from monocultural families. This shows that adolescents from multicultural families experienced mood modifications like stress relief and a temporary escape through online games relatively more than adolescents from monocultural families.

In the case of the “problem” factor referred as problems caused by excessive game play (e.g., “Did you feel bad after playing for a long time”), only adolescents from monocultural/single-income families showed a significantly lower average value than the other groups. This means that adolescents from monocultural/single-income families, who were free from both the multicultural family and dual-income family factors (selected as the independent variables in this study), were less likely to experience the “problem” factor than other groups.

Previous studies have shown that children from multicultural families often experience group bullying because of their exotic appearance [51]. Continuation of such isolation can lead to a decrease in self-competence [52] and social avoidance tendencies [53]. Eventually, those with poor interpersonal relationships are more likely to escape reality and become addicted to the internet [54] and go on to become addicted to online games [55]. In addition to the factors mentioned above, previous studies have shown that children are more immersed in internet use and online games when their parents work long hours due to dual-careers [8].

The results of the present study are largely similar to previous research. This is because adolescents are not only more influenced by external contextual factors than adults but also, to some extent, lack the ability to control their own needs [56]. In this context, these results will contribute to the data for adolescent education, as they indicate that the most important factors in adolescent game addiction tendency, which has recently emerged as a social issue, are derived from society or their parents, rather than being their own fault.

### 5.2. Delinquency/Deviance due to Differences in Family Environment

In this study, being from a multicultural family was not a significant factor concerning juvenile delinquency. However, the dual-income family environment was found to be a critical factor in juvenile delinquency. Specifically, adolescents from dual-income families scored significantly higher on all factors including smoking, drinking, and school absence compared to those from single-income families. This result was consistent with the previous literature that dual-income families tend to have problems regarding child-rearing in spite of a better financial status [4].

Ashford and Lecroy [57] argued that the main characteristics of juvenile delinquents included more rebelliousness, more hostility, and poorer self-regulatory abilities compared with typical adolescents. Among the important factors of adolescent engagement in delinquent behaviors, the role of parents has been reported to be particularly important [58]. Therefore, parental interest toward children, and the total amount of time spent with them seem to be critical in reducing juvenile delinquency, rather than invisible social discrimination or prejudice toward children from multicultural families. In particular, considering that, the factors related to addiction, “salience”, “tolerance” and “withdrawal” were found to be significant predictors of juvenile delinquency, it is apparent that the role of parents is very important in addition to adolescents’ self-control.

### 5.3. Participation Motivation due to Differences in Family Environment

The MANOVA showed statistically significant differences between the groups in “pass time” and “social interaction”. Follow-up analysis found that monoculturalism/multiculturalism had no effect on “pass time” but dual-income/single-income did, meaning that adolescents in dual-income families were motivated to play online games to pass the time. This suggests that adolescents in dual-income families whose parents are not at home after school tend to send more time to play online games, which is consistent with results of previous literature [14].

There was no effect of dual-/single-income on “social interaction” but there was an effect from multi/monoculturalism, suggesting that adolescents in multicultural families play online games to engage in social interaction. This result suggests that adolescents in multicultural families pursue social interaction in the online environment characterized by anonymity.

In conclusion, adolescents from dual-income families showed a tendency to rely on online games when alone [6], and those from multicultural families participated in online games for self-expression [59]. In addition, while there were no significant differences between groups, “entertainment”, “competition” and “arousal” had higher mean scores than other factors, which suggests that, regardless of family types, adolescents generally enjoy online games with the motives of “entertainment”, “competition” and “arousal” characteristics of such games.

## 6. Conclusions

This study focused on the family form (double-income and multicultural family) that is changing in Asian countries due to various reasons, just like in their Western counterparts. In other words, this study compared and analyzed how such changes in family form have an impact on online game addiction and delinquency in children who are going through their sensitive adolescent stage. Further, we conducted an empirical examination of the significant cause-and-effect relationship between online game addiction and delinquency in adolescents. As mentioned above, this study interestingly found that not only does this new family form have a considerable impact on adolescents, but also that online game addition increases delinquent behaviors. This is a significant finding that implies the need for more focus and training on adolescents, as they have not yet grown into adults. Specifically, it is necessary to provide programs for afterschool care and education by employing helpers for dual income families at the school level or establishing systems to prevent indiscreet game addiction among adolescents by limiting the hours of use and ensuring online activity occurs under their real names. Moreover, this study will serve useful for developing efficient social welfare plans by the Education Ministry and the government.

There are several limitations regarding external validity and research design. This study was conducted in South Korea, a country at a top global rank in terms of internet communication network supply rate and average speed. Therefore, while it is true that Korean adolescents play several online games, it is necessary to see the results for adolescents in other Asian countries where the internet is not yet widely commercialized. Since the preferences and characteristics of adolescents may differ according to their country, even if they are from the same continent, a more in-depth follow-up study must be conducted on these differences. Also, if an in-depth interview or other qualitative research method is implemented, it will be a good opportunity to solve the social and psychological issues of future adolescents on the basis of more diverse opinions. Moreover, this study has limitations that prevent generalizing the results to other Asian countries because the research was conducted exclusively in Korea. Accordingly, for follow-up research, an in-depth study must be conducted based on comparative analyses of Asian countries. Lastly, the cross-sectional data may not support cause and effect relationships in this study conducted for a relatively short period of time. Thus, a future research with longitudinal study design will be able to capture mentality and behaviors of adolescents.

## Figures and Tables

**Table 1:** Frequency of distributions for socio-demographic variables.

Variables	Categories	Frequency (*n* = 246)	Percentage (100%)
Gender	Male Female	202 44	82 18
Culture	Single	131	53
	Multiple	115	47
Income	Single	133	54
	Double	113	46

**Table 2:** Results of multiple regression: effects of online game addiction on juvenile delinquency.

DV	IV	*B*	*β*	*t*	*p*	*R* ^2^	*F*	*p*
Juvenile Delinquency						0.20	8.559	0.000 ***
	Salience	0.30	0.32	5.24	0.000 ***			
	Withdrawal	0.19	0.18	3.02	0.003 **			
	Tolerance	0.12	0.12	2.01	0.045 *			

*Note.* * *p* < 0.05, ** *p* < 0.01, *** *p* < 0.001.

**Table 3:** Results of MANOVA: Differences in addiction in esports between four groups based on the forms of families.

Source	DV	*df*	*F*	*p*	*η* ^2^
Addiction	Salience	3	24.394	0.000 ***	0.232
	Tolerance	3	15.265	0.000 ***	0.159
	Mood modification	3	9.126	0.000 ***	0.102
	Relapse	3	1.333	0.264	0.016
	Withdrawal	3	17.171	0.000 ***	0.176
	Conflict	3	1.213	0.306	0.015
	Problem	3	8.052	0.000 ***	0.091

*Note.* *** *p* < 0.001.

**Table 4:** Mean scores for addiction in esports among groups and scale reliability.

	Salience	Tolerance	Mood Modification	Relapse	Withdrawal	Conflict	Problem
Group1	1.83	1.82	1.85	2.91	1.69	1.80	1.69
Group2	**2.96**	**2.69**	1.85	2.97	**2.40**	1.79	**2.32**
Group3	1.82	1.77	**2.46**	2.83	1.71	1.90	**2.41**
Group4	**2.69**	**2.52**	**2.47**	3.15	**2.56**	1.62	**2.22**

*Note.* Group1 = Single cultural/Single income, Group2 = Single cultural/Double income, Group3 = Multi cultural/Single income, Group4 = Multi cultural/Double income. Statistically significant higher mean scores between groups in bold.

**Table 5:** Result of ANOVA, mean scores, and scale reliability: Differences in juvenile delinquency in esports between four groups based on the forms of families.

	SS	*Df*	*MS*	*F*	*p*
Between Groups	89.280	3	29.760	48.100	0.000 ***
Within Groups	149.728	242	0.619		
Total	239.008	245			
		**Group1**	**Group2**	**Group3**	**Group4**
Juvenile Delinquency		1.62	**2.97**	1.70	**2.73**

*Note.* *** *p* < 0.001. Group1 = Single cultural/Single income, Group2 = Single cultural/Double income, Group3 = Multi cultural/Single income, Group4 = Multi cultural/Double income. Statistically significant higher mean scores between groups in bold.

**Table 6:** Results of MANOVA: Differences in participation motivations in esports between four groups based on the forms of families.

Source	DV	*df*	*F*	*p*	*η* ^2^
Participation	Entertainment	3	0.972	0.407	0.012
Motivation	Competition	3	1.952	0.122	0.024
	Permanence	3	2.608	0.052	0.031
	To pass time	3	40.703	0.000 ***	0.335
	Social interaction	3	28.530	0.000 ***	0.261
	Diversion	3	1.789	0.150	0.022
	Arousal	3	0.331	0.803	0.004
	Peer pressure	3	0.069	0.976	0.001

*Note.* ***p < 0.001.

**Table 7:** Mean scores for participation motivations in esports among groups and scale reliability.

	1	2	3	4	5	6	7	8
Group1	4.91	4.79	3.67	3.21	3.16	3.98	4.48	3.92
Group2	4.63	4.82	3.47	**4.97**	3.07	3.88	4.32	3.92
Group3	4.77	4.40	3.15	3.34	**4.34**	3.96	4.29	3.95
Group4	4.54	4.45	3.12	**4.97**	**4.77**	4.44	4.46	3.84

*Note.* Group1 = Single cultural/Single income, Group2 = Single cultural/Double income, Group3 = Multi cultural/Single income, Group4 = Multi cultural/Double income. 1 = Entertainment, 2 = Competition, 3 = Permanence, 4 = To pass time, 5 = Social interaction, 6 = Diversion, 7 = Arousal, 8 = Peer pressure. Statistically significant higher mean scores between groups in bold.

## References

[1] Jo H.Y., Seo D.H., Kwon S.H. (2008). An ethnographic study on the academic performance of children of migrants. Korean J. Soc. Educ..

[2] Ministry of the Interior and Safety (2009). Employment Extension of Public Officer for Foreigners and North Korean Defectors. http://www.mois.go.kr/frt/bbs/type010/commonSelectBoardArticle.do?bbsId=BBSMSTR_000000000008&nttId=26822.

[3] Kim B.S. (2008). An ethnographic study on the kindergarten life of the children of multicultural family. Res. Cent. Korean Youth Cult..

[4] Moon H.J., Choi Y.K., Seo S.J. (2009). Children in Korean multi-cultural families. Korean Assoc. Child Stud..

[5] Choi Y.J., Choi M., Choi S.B. (2018). The effect of dual earner couple’s role assignment on work-family conflict, family life satisfaction, and depression. J. Soc. Res..

[6] Lee J.H., Lee E.H. (2000). The moderating effect of coping strategies upon multiple role conflicts and depression within dual employed couples. Korean J. Health Psychol..

[7] Peterson G.W., Hennon C.B., McKenry P.C., Price S.J. (2005). Conceptualizing parenting stress with family stress theory. Families and Change.

[8] Pἅἅkkὅnen H. (2008). Alone at home. Int. J. Time Use Res..

[9] King S.A. Is the Internet Addictive, or Are Addicts Using the Internet?. 1996..

[10] Brenner V. (1997). Psychology of computer use: XLVII. Parameters of Internet use, abuse and addiction: The first 90 days of the Internet Usage Survey. Psychol. Rep..

[11] Hauge M.R., Gentile D.A. Video game addiction among adolescents: Associations with academic performance and aggression. Proceedings of the Society for Research in Child Development Conference.

[12] Young K.S. (1996). Internet addiction: The emergence of a new clinical disorder. Cyberpsychol. Behav..

[13] Kim Y.E., Park S.H. (2007). A study on the online game use influences in game flow and addiction: Focusing on the uses and gratifications approach. Korean J. Commun. Stud..

[14] Kim D. (2012). An analysis on the difference of juvenile delinquency by online-game addiction. Korean Crim. Psychol. Res..

[15] Noh S.-J. (2010). Analyzing socio-psychological factors affecting internet addiction in university students. J. Korean Educ..

[16] Bányai F., Griffiths M.D., Király O., Demetrovics Z. (2018). The psychology of esports: A systematic literature review. J. Gambl. Stud..

[17] Taylor T. (2012). Raising the Stakes: E-Sports and the Professionalization of Computer Gaming.

[18] Xu Z., Turel O., Yuan Y. (2012). Online game addiction among adolescents: Motivation and prevention factors. Eur. J. Inf. Syst..

[19] Choi S.W., Kim H.S., Kim G.Y., Jeon Y., Park S.M., Lee J.Y., Jung H.Y., Sohn B.K., Choi J.S., Kim D.J. (2014). Similarities and differences among Internet gaming disorder, gambling disorder and alcohol use disorder: A focus on impulsivity and compulsivity. J. Behav. Addict..

[20] Lemmens J.S., Valkenburg P.M., Peter J. (2009). Development and validation of a game addiction scale for adolescents. Media Psychol..

[21] Wei H.T., Chen M.H., Huang P.C., Bai Y.M. (2012). The association between online gaming, social phobia, and depression: An internet survey. BMC Psychiatry.

[22] Loton D., Borkoles E., Lubman D., Polman R.C.J. (2016). Video game addiction, engagement and symptoms of stress, depression and anxiety: The mediating role of coping. Int. J. Ment. Health Addict..

[23] Guo J., Chen L., Wang X., Liu Y., Kwan C.H., He H., Qu Z., Tian D. (2012). The relationship between Internet addiction and depression among migrant children and left-behind children in China. Cyberpsychol. Behav. Soc. Netw..

[24] Young K., Pistner M., O’Mara J., Buchanan J. (2000). Cyber-Disorders: The mental health concern for the new millennium. Cyberpsychol. Behav..

[25] Grüsser S.M., Tahlemann R., Griffiths M.D. (2007). Excessive computer game playing: Evidence for addiction and aggression?. Cyberpsychol. Behav..

[26] Lo S., Wang C., Fang W. (2005). Physical interpersonal relationships and social anxiety among online game players. Cyberpsychol. Behav..

[27] Ko C.N., Liu T.L., Wang P.W., Chen C.S., Yen C.F., Yen J.Y. (2014). The exacerbation of depression, hostility, and social anxiety in the course of internet addiction among adolescents: A prospective study. Compr. Psychiatry.

[28] Beranuy M., Carbonell X., Griffiths M. (2013). A qualitative analysis of online gaming addicts in treatment. Int. J. Ment. Health Addict..

[29] Henchoz Y., Studer J., Deline S., N’Goran A.A., Baggio S., Gmel G. (2016). Video gaming disorder and sport and exercise in emerging adulthood: A longitudinal study. Behav. Med..

[30] Griffiths M.D., Davies M., Chappell D. (2004). Online computer gaming: A comparison of adolescent and adult gamers. J. Adolesc..

[31] Lee H.J. (2014). The effects of social supports and strains on adolescent delinquency. Master’s Thesis.

[32] Senna J., Siegel L. (2014). Juvenile Delinquency: Theory, Practice and Law.

[33] Cho Y.S., Shin Y.S. (2001). Causal pathway analysis of adolescence status delinquency. J. Korean Acad. Soc. Nurs. Educ..

[34] Robert A. (2005). Desisting from crime: Continuity and change in long-term crime patterns of serious chronic offenders. Contemp. Crisis.

[35] Moon J.W. (2012). Factors affecting runaway impulse and experience of adolescents. Korean Public Health Res..

[36] Kuss D.J., Griffiths M.D. (2012). Online gaming addiction in children and adolescents: A review of empirical research. J. Behav. Addict..

[37] Kim E.J., Kee N., Ku T., Kim S.J. (2008). The relationship between online game addiction and aggression, self-control and narcissistic personality traits. Eur. Psychiatry.

[38] Anderson C., Sakamoto A., Gentile D., Ihori N., Shibuya A., Yukawa S. (2008). Longitudinal effects of violent video games on aggression in Japan and the United States. Pediatrics.

[39] Etchells P.J., Gage S.H., Rutherford A.D., Munafò M.R. (2016). Prospective investigation of video game use in children and subsequent conduct disorder and depression using data from the Avon longitudinal study of parents and children. PLoS ONE.

[40] Kutner L., Olson C. (2008). Grand Theft Childhood: The Surprising Truth About Violent Video Games and What Parents Can Do.

[41] Ko Y.J., Park H., Claussen C.L. (2008). Action sports participation: Consumer motivation. Int. J. Sports Mark. Spons..

[42] Shank M.M. (1998). Fan or fanatic: Refining a measure of sports involvement. J. Sport Manag..

[43] Rubin A.M., Bryant I.J., Zillmann D. (1994). Media uses and effects: A uses and gratifications perspective. Media Effects: Advances in Theory and Research.

[44] Casselman B. (2015). Resistance Is Futile: eSports Is Massive… and Growing. http://www.espn.com/espn/story/_/id/13059210/esports-massive-industry-growing.

[45] Lee D., Schoenstedt L.J. (2011). Comparison of eSports and traditional sports consumption motives. ICHPER-SD J. Res..

[46] Stanford Children’s Care. https://www.stanfordchildrens.org/en/topic/default?id=the-growing-child-adolescent-13-to-18-years-90-P02175.

[47] WHO. http://www.who.int/maternal_child_adolescent/topics/adolescence/development/en/.

[48] Kim Y., Ross S.D. (2006). An exploration of motives in sport video gaming. Int. J. Sports Mark. Spons..

[49] Sherry J.L., Lucas K., Greenberg B.S., Lachlan K., Vorderer P., Bryant J. (2006). Video game uses and gratifications as predictors of use and game preference. Playing Video Games: Motives, Responses, and Consequences.

[50] Nunnally J.C., Bernstein I.H. (1994). Psychometric Theory.

[51] Seol D.H. (2006). International marriage migration and national identity: Marriage-based immigrants’ and their children’s self-identification of Koreanness with regard to ethnic and civic nationhood in South Korea. Econ. Soc..

[52] Kurdek L.A., Krile D. (1982). A developmental analysis of the relation between peer acceptance and both interpersonal understanding and perceived social self-competence. Child Dev..

[53] Asher S.R., Parkhurst J.T., Hymel S., Williams G.A., Asher A.R., Coie J.D. (1990). Peer rejection and loneliness in childhood. Peer Rejection in Childhood.

[54] Young K.S., Van de Creek L., Jackson X. (1997). Internet addiction: Symptoms, evaluation and treatment. Innovations in Clinical Practice: A Source Book.

[55] Chak K., Leung L. (2004). Shyness and locus of control as predictors of internet addiction and internet use. CyberPsychol. Behav..

[56] Gupta R., Derevensky J.L. (2000). Adolescents with gambling problems: From research to treatment. J. Gambl. Stud..

[57] Ashford J.B., LeCroy C.W. (1990). Juvenile recidivism: A comparison of three prediction instruments. Adolescence.

[58] Steinberg L., Silverberg S.B. (1986). The vicissitudes of autonomy in early adolescence. Child Dev..

[59] Park S.B., Chung N.H. (2010). A factor affecting self-presentation desire and participation intention in online game. e-Bus. Stud..

